# Dysconnectivity of neurocognitive networks at rest in very-preterm born adults^☆^

**DOI:** 10.1016/j.nicl.2014.01.005

**Published:** 2014-01-18

**Authors:** Thomas P. White, Iona Symington, Nazareth P. Castellanos, Philip J. Brittain, Seán Froudist Walsh, Kie-Woo Nam, João R. Sato, Matthew P.G. Allin, Sukhi S. Shergill, Robin M. Murray, Steve C.R. Williams, Chiara Nosarti

**Affiliations:** a Department of Psychosis Studies, Institute of Psychiatry, King's College London, de Crespigny Park, London SE5 8AF, UK; b Centre of Mathematics, Computation and Cognition, Universidade Federal do ABC, Av. dos Estados, 5001 Bairro Bangu, Santo André, SP CEP 09210-580, Brazil; c Department of Neuroimaging, Institute of Psychiatry, King's College London, de Crespigny Park, London SE5 8AF, UK

**Keywords:** Preterm birth, Resting-state, Functional connectivity, Neurocognitive networks, Executive function

## Abstract

Advances in neonatal medicine have resulted in a larger proportion of preterm-born individuals reaching adulthood. Their increased liability to psychiatric illness and impairments of cognition and behaviour intimate lasting cerebral consequences; however, the central physiological disturbances remain unclear. Of fundamental importance to efficient brain function is the coordination and contextually-relevant recruitment of neural networks. Large-scale distributed networks emerge perinatally and increase in hierarchical complexity through development. Preterm-born individuals exhibit systematic reductions in correlation strength within these networks during infancy. Here, we investigate resting-state functional connectivity in functional magnetic resonance imaging data from 29 very-preterm (VPT)-born adults and 23 term-born controls. Neurocognitive networks were identified with spatial independent component analysis conducted using the Infomax algorithm and employing Icasso procedures to enhance component robustness. Network spatial focus and spectral power were not generally significantly affected by preterm birth. By contrast, Granger-causality analysis of the time courses of network activity revealed widespread reductions in between-network connectivity in the preterm group, particularly along paths including salience-network features. The potential clinical relevance of these Granger-causal measurements was suggested by linear discriminant analysis of topological representations of connection strength, which classified individuals by group with a maximal accuracy of 86%. Functional connections from the striatal salience network to the posterior default mode network informed this classification most powerfully. In the VPT-born group it was additionally found that perinatal factors significantly moderated the relationship between executive function (which was reduced in the VPT-born as compared with the term-born group) and generalised partial directed coherence. Together these findings show that resting-state functional connectivity of preterm-born individuals remains compromised in adulthood; and present consistent evidence that the striatal salience network is preferentially affected. Therapeutic practices directed at strengthening within-network cohesion and fine-tuning between-network inter-relations may have the potential to mitigate the cognitive, behavioural and psychiatric repercussions of preterm birth.

## Highlights

• Functional connectivity was investigated using fMRI in preterm-born adults at rest.
• Generalised partial directed coherence was assessed between neurocognitive networks.
• Preterm-born adults exhibited widespread reductions in connection strength.
• Coherence-derived graph topology permitted identification of preterm-born adults.

## 1 Introduction

Infants born at or before a gestational age of 33 weeks (very-preterm; VPT) are likely to exhibit cognitive, educational and behavioural problems in childhood (Boyle et al., 2011; Johnson and Marlow, 2011), which persist through adolescence into adulthood (Allin et al., 2008; Hack, 2009; Hille et al., 2007; Saigal and Doyle, 2008). Preterm birth has further been associated with an increased risk of psychiatric disorders (Boyle et al., 2011; Dalziel et al., 2007; Hack et al., 2004; Nosarti et al., 2012; Walshe et al., 2008; Wiles et al., 2005).

Our understanding of the cerebral sequelae of VPT-birth is considerable for early stages of life. The brains of VPT-born infants often exhibit haemorrhagic and hypoxic–ischaemic damage leading to ventricular dilatation, white matter abnormality, an enlarged subarachnoid space (Inder et al., 2003) and regional myelin damage (Sie et al., 1997). Further, it appears that rates of cortical growth and related microstructure development are reduced during infancy in line with prematurity (Ball et al., 2013). Anatomical abnormalities observed during childhood include hemispherically-asymmetric reductions in grey-matter volume in temporal and peri-Rolandic cortex (Peterson et al., 2000), increases in parietal and frontal cortices which are predicted by gestational age at birth (Kesler et al., 2004); and bilateral increases in gyrification in the temporal cortex (Kesler et al., 2006). Together these findings suggest widespread disruption to prototypic patterns of cerebral development. Similarly, in adolescence, intricate and distributed anomalies of grey-matter volume are exhibited by VPT-born individuals. In addition to volume decreases in regions including the frontal, temporal, insular and occipital cortices, caudate nucleus and putamen, increases are also seen in frontal and temporal regions, cerebellum and cingulate gyrus (Nosarti et al., 2008). These findings are compatible with a systematic and functionally-influential structural remodelling of cerebral architecture in association with premature birth (Allin et al., 2010). Consistent with the anatomical literature are reports that blood oxygenation-level dependent (BOLD) activation responses during language, inhibitory control, attention allocation and associative learning imply that VPT-born adolescents adopt possibly compromised and alternative functional pathways (Gimenez et al., 2005; Kalpakidou et al., 2012; Lawrence et al., 2009; Peterson et al., 2002).

According to Hebbian principles, it is likely that these observed disturbances of neuronal infrastructure are at least in part functionally driven. Since both endogenous and event-related neural activity is vital for the refinement of nervous-system circuitry (Penn and Shatz, 1999), the emergence of neural networks is likely to play a critical role in cortical development; and related alteration is implied in the preterm brain. The study of resting-state functional connectivity (RSFC) has demonstrated intrinsic and coherent BOLD signal fluctuations in dissociable networks involving large-scale, spatially-disparate regions (Damoiseaux et al., 2006). The functional relevance of these resting-state networks (RSNs) can be inferred from their spatial congruence with networks of regions co-activated in association with specific cognitive and sensory tasks (Biswal et al., 1995; Fox et al., 2005; Hampson et al., 2002); as well as inter-region anatomical pathways (Greicius et al., 2009; Skudlarski et al., 2008; Vincent et al., 2007).

An important development was marked by recent studies of the ontological development of RSNs. In a pioneering study, Fransson et al. (2007) demonstrated that RSNs are evident at term-equivalent age (around 40 weeks of gestation) in VPT-born infants, albeit with lesser complexity than adult networks; term-equivalent networks are fewer in number, non-lateralised and restricted to hemispherically-homologous structures. Doria et al. (2010) further demonstrated that even complex RSNs, such as the executive control network, are also present at term, and before the acquisition of related cognitive functions later in childhood. Supekar et al. (2009) complementarily observed that the hierarchical organisation of RSNs increases from childhood to young adulthood; and suggested that whilst highly-hierarchical brains afford benefits in terms of maximising rapid top-down communication and minimising wiring costs in adults, the less-hierarchical arrangement in children may serve to protect from the potential of pervasive effects resulting from damage to hubs. Together, these findings illustrate the non-stationarity of RSNs through development.

There is direct evidence that preterm birth is associated with RSN abnormality. Smyser et al. (2011) investigated RSFC in VPT- and term-born infants using seed-region connectivity and across wide-ranging seeds found consistently lower correlations, a more limited distribution of significant correlation and decreased long-range functional connectivity in VPT-born infants. Furthermore, longitudinal investigation in the same group revealed age-dependent increases in correlation strength. These findings demonstrate the advantageous effects of RSN development in the sheltered uterine environment during the final gestational weeks; and are consistent with structural observations that there are significant increases in long-range cortico-cortical connectivity and noteworthy resolution of distinct cortical lamina in this period (Kostovic and Jovanov-Milosevic, 2006).

Since large cohorts of VPT-born individuals are now reaching adulthood, in light of substantial improvements in neonatal care over the past three decades, it has become more feasible to investigate the lasting effects of VPT-birth on the adult brain. Furthermore, in view of the observed effects on infant brains, study of RSFC in VPT-born adults is strongly warranted. Here, we apply spatial independent component analysis (ICA) (Bell and Sejnowski, 1995) – a data-driven blind-source separation method that identifies spatial patterns on the basis of their maximal spatial independence – to resting-state fMRI data collected in VPT-born adults and in term-born controls. In line with Menon's (2011) unifying triple-network model of psychopathology and the established cognitive impairments demonstrated by preterm-born adults, this investigation is focused on three neurocognitive RSNs: the default mode network (DMN) (Raichle et al., 2001), which comprises the medial prefrontal, posterior cingulate, precuneus and bilateral angular gyrus, and whose activity is elevated during introspective tasks (Gusnard et al., 2001); the central executive network (CEN), which includes dorsolateral prefrontal and parietal regions, and is activated by tasks involving externally-focused attention, working memory and response selection (Corbetta et al., 2002; Hester et al., 2007; Meda et al., 2008); and the salience network (SN), which is focused in frontoinsular cortex, dorsal anterior cingulate cortex (ACC) and subcortical structures including the striatum (Seeley et al., 2007), and whose activity is driven by cognitive, emotional or homeostatic salience.

The principal aims of this work are threefold: first, to investigate whether VPT birth is associated with systematic changes in the spatial and spectral characteristics of these large-scale neurocognitive networks in early adulthood; second, to evaluate whether the causal relationships between the time courses of these networks are altered in VPT-born compared to term-born individuals using Granger causality; and third – in order to test the potential clinical relevance of these indices – to examine the extent to which these network-focused measures are associated with functional alterations observed in VPT samples.

## 2 Method

### 2.1 Participants

29 VPT-born individuals (< 33 weeks of gestation) were recruited to take part in this study from a large cohort of individuals who had been admitted to the Neonatal Unit at University College Hospital, London between 1979 and 1984, who have been followed up to investigate the long-term consequences of premature birth. VPT-born participants' neonatal ultrasound (US) scans were classified as: normal (n = 16); exhibiting uncomplicated periventricular haemorrhage (n = 4); or exhibiting periventricular haemorrhage and ventricular dilatation (n = 9). Please refer to Nosarti et al. (2008) for further details on US classification.

23 age-matched term-born individuals (37–42 weeks of gestation) were also assessed. The inclusion criterion for this group was full-term birth (37–42 completed weeks of gestation); exclusion criteria were birth complications (for example, low birth weight defined as < 2500 g, endotracheal mechanical ventilation, prolonged gestation (> 42 weeks)).

Exclusion criteria for all participants were severe hearing and motor impairment, or any mental retardation. Two participants from each group had a personal history of psychiatric illness (VPT-born group: bipolar disorder (n = 1); borderline personality disorder (n = 1); term-born group: depression (n = 2)). One member of the term-born group was prescribed an antidepressant medication (sertraline) at time of study. All participants were English native speakers. All participants gave informed written consent and were reimbursed for travel expenses and received a nominal remuneration for participation in the study. The study was given ethical approval by the South London and Maudsley Research and Ethics Committee and by the Psychiatry, Nursing and Midwifery Research Ethics Subcommittee (PNM RESC), King's College London.

Intelligence quotient (IQ) was assessed for each individual by a rater blind to group membership using the Wechsler Abbreviated Scale of Intelligence (WASI) (Wechsler, 1999); and the Hayling sentence completion test (Burgess and Shallice, 1997) was used to measure executive function. Table 1 displays sample characteristics for both study groups. Chi-squared testing demonstrated that the groups did not differ by sex. Independent sample T-tests showed non-significant between-group differences in age and IQ, although the VPT-born group exhibited significantly reduced executive function scores (Hayling scaled values) as compared with the term-born individuals (T(46) = 2.12, P = .032). Within the VPT-born group, a Spearman's rank test performed to investigate the relationship between gestational age (GA) at birth and neonatal US classification (coded as normal (0), uncomplicated periventricular haemorrhage (1), and periventricular haemorrhage and ventricular dilatation (2)) revealed a significant negative relationship between these perinatal risk factors (Spearman's ρ = − .545, P = .002).

### 2.2 fMRI data collection

256 gradient-echo echo-planar images (TR/TE: 2000/30 ms, flip angle: 75°, matrix: 64 × 64) were acquired on a 3 Tesla GE Signa MR scanner (GE Healthcare, USA) at the Maudsley Hospital, London. Each whole-brain image contained 37 non-contiguous slices of 2.4-mm thickness separated by a distance of 1 mm, and with in-plane isotropic voxel resolution of 3.4 mm. Participants were instructed to remain still with gaze fixed on a central cross for the duration of this nine-minute resting-state scan.

### 2.3 fMRI preprocessing and independent component analysis

fMRI data were preprocessed using SPM8 (Statistical Parametric Mapping, Wellcome Department of Imaging Neuroscience, University of London, UK). Data were spatially realigned to the first image of the series, time-corrected to the first slice of each image, normalised to a standard template of the Montreal Neurological Institute (MNI) brain, and smoothed using an 8-mm full-width at half-maximum Gaussian kernel.

Spatial ICA was performed on the preprocessed data using the Group ICA fMRI Toolbox (GIFT; http://icatb.sourceforge.net) within MATLAB 7.8 (MathWorks, USA). GIFT uses a temporal concatenation approach, during which data reduction is performed via multi-staged principal component analysis and aggregation to generate common-group maps. These components are then back-projected onto each individual's data to create subject-specific spatial maps with corresponding time courses (Calhoun et al., 2008; Calhoun et al., 2009).

Prior to ICA, data dimensionality was estimated (using Minimum Description Length criteria) to be 44. Since model-order determines network spatial characteristics including subnetwork parcellation, ICA was constrained to produce 44 components. ICA was performed using the Infomax algorithm (Bell and Sejnowski, 1995), and repeated 5 times with Icasso (Himberg et al., 2004) to maximise the stability of the derived components. Components were also scaled according to percent signal change to facilitate inter-subject comparisons of their time courses. Back-reconstruction was carried out using GICA3 on the basis of previous empirical support for the accuracy of this method (Erhardt et al., 2011).

### 2.4 Between-group differences in head motion

In light of recent reports that head motion can influence patterns of intrinsic functional connectivity as measured by fMRI (Power et al., 2012), possible systematic between-group differences in head motion in the current dataset were calculated using the techniques set out by van Dijk et al. (2012), which examine the movement parameters calculated during realignment. Four metrics were calculated for each study participant: mean motion; maximum motion; number of movements; and rotation. Mean motion represented the mean absolute displacement of each brain volume as compared with its preceding volume and used the translation parameters in the x, y and z planes. Displacement was computed as the root mean square of these values. Maximum motion was the greatest displacement value across the dataset. Number of movements was calculated by evaluating the number of brain volumes in which displacement exceeded 0.1 mm, and was thus bounded between a minimum value of 0 and a maximum value of n − 1 where n was the number of brain volumes in the time series. Rotation was calculated according to Euler's rotation theorem that expresses any three-dimensional rotation as a single angle and corresponding axis of rotation, and computed for each brain volume in comparison with its preceding volume. The Euler angle was computed using the following formula: arccos((cos(phi)cos(theta) + cos(phi)cos(psi) + cos(theta)cos(psi) + sin(phi)sin(psi)sin(theta) − 1) / 2), where phi, theta, and psi are the respective rotational parameters around the x, y and z axes. Between-group differences in these metrics were assessed using independent sample T-tests on 1000 bootstrapped samples, with the α-threshold for the bootstrapped P-value Bonferroni corrected to .0125 to reflect the examination of the four head-motion characteristics. The approach of combining leave-one-out permutation with Bonferroni correction of the significance threshold was used where feasible throughout this work as a means of enhancing the dependability of reported findings.

### 2.5 Component selection

**Fig. 1:**
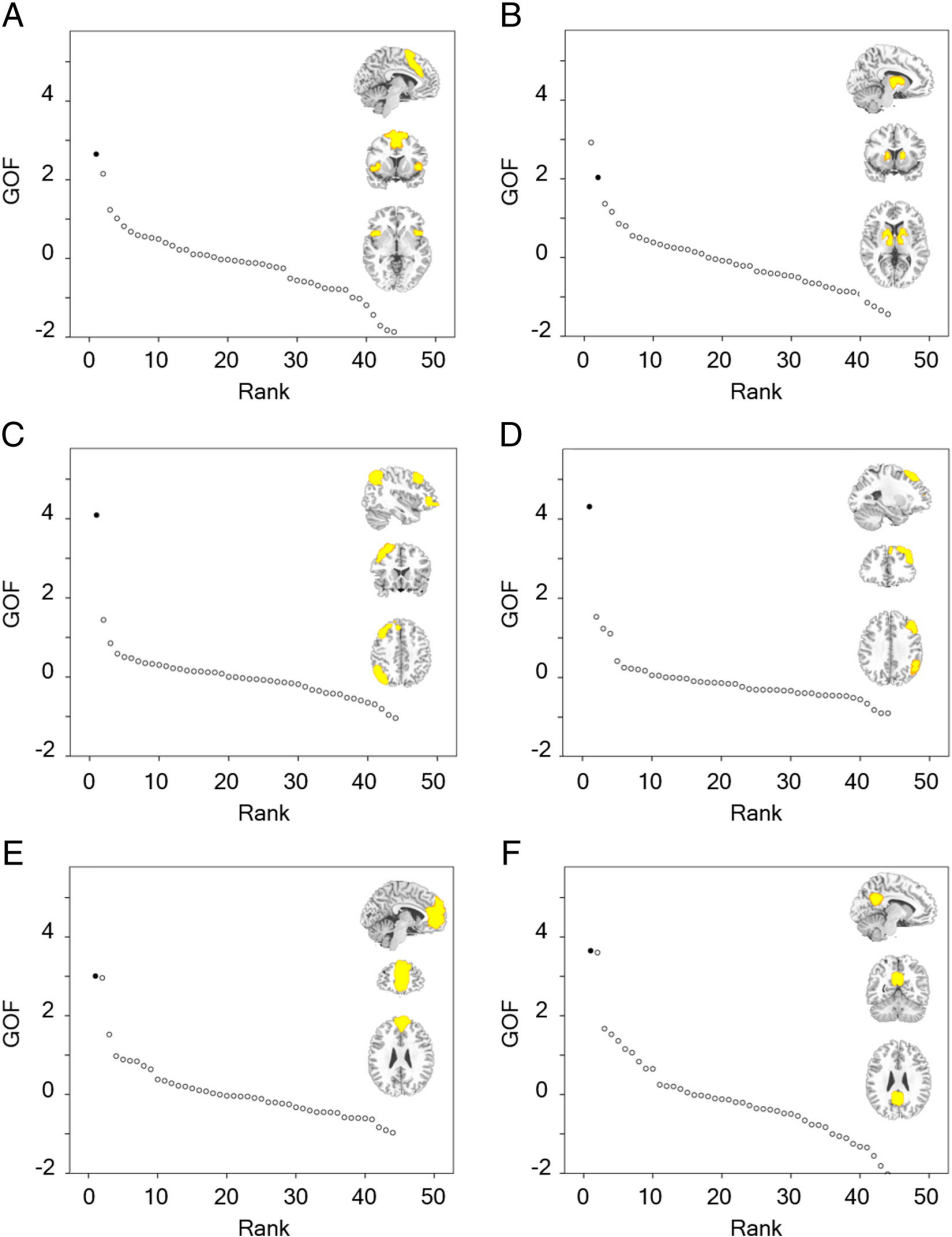
Ranked goodness-of-fit (GOF) scores for each component with pre-specified functionally-derived masks, for: (A) insular salience network; (B) striatal salience network; (C) left central executive network; (D) right central executive network; (E) frontal default mode network; and (F) posterior default mode network. In each sub-figure, the component chosen for subsequent extended analysis is depicted by a black circle and all other components are represented by white circles. The insets depict the binary mask for each network in yellow overlaid on sections of a standardised T1-weighted image.

A goodness-of-fit (GOF) procedure was used to assess the spatial correspondence between each of the 44 whole-sample component maps and binary masks constructed from previous work to characterise features of the three networks upon which this work is focused (http://findlab.stanford.edu/research; Shirer et al., 2012). Each network was split into two subnetworks to permit investigation of region-specific phenomena. These masks are depicted in Fig. 1. The salience network masks comprised: (i) bilateral anterior insula and dorsal anterior cingulate cortex; and (ii) basal ganglia regions. It is reasonable to dissociate the latter on account of the fundamental role that the striatum is believed to play in salience assignment and the inclusion of striatal and thalamic structures in the original description of this network (Seeley et al., 2007). The CEN masks split the CEN into its left and right hemispheric nodes (as per previous fMRI investigations of this network; Sridharan et al., 2008; White et al., 2010). The DMN masks included: (i) the anterior medial prefrontal cortex node of the DMN; and (ii) the posterior midline structures of the posterior cingulate gyrus and precuneus. Again, these sub-features were investigated independently in the current study on the basis of their division in previous ICA investigations (for example, Sridharan et al., 2008; White et al., 2010). GOF scores were calculated by Z-scoring each component map and then subtracting the mean voxel value outside a binary mask from the mean voxel value within it (Seeley et al., 2007; White et al., 2013). Component selection was performed according to ranked GOF scores.

One sample T-tests were conducted on back-reconstructed images of component loadings for each component of interest to assess component spatial robustness within the sample as a whole. Clusters of significant positivity were ascribed for each component using a family-wise error corrected P-threshold of .05 on the basis of the number and spatial extent of significant voxels at an uncorrected P-threshold of .001 (Friston et al., 1994).

### 2.6 Spatial differences between VPT-born and term-born group components

To assess between-group differences in the whole-brain spatial focus, extent and amplitude of each component, two sample T-tests were conducted on the back-reconstructed component loading maps. Effects were investigated in all six components independently and evaluated across the whole brain. As in the previous analysis, the significance of between-group differences was ascribed according to a voxel-level inclusion of P < .001 uncorrected and a cluster-level significance of P < .05 family-wise error corrected (Friston et al., 1994).

### 2.7 Assessing between-group differences in component power spectra

To investigate between-group differences in the spectral characteristics of activity, power spectral density (PSD) was evaluated for the individual-specific time courses for the six components of interest. Mean PSD was then calculated between 0.05 and 0.15 Hz in line with subsequent Granger-causality measures. For each component a permutation analysis was conducted in which between-group effects were tested using multiple Kruskal–Wallis tests and a leave-one-out strategy for all possible combinations of participants, with between-group effects judged significant in this analysis if reliable in more than 99.17% of permutations. This threshold represents Bonferroni-correction of the conventional 95% threshold on account of the six tests performed. Kruskal–Wallis tests were used as a non-parametric alternative here and in all other analyses where skewness and kurtosis of the relevant data features precluded the assumption of normality. Cut-offs of 2 and 7 were used for skewness and kurtosis respectively (Curran et al., 1996).

### 2.8 Granger-causality analysis and classification

Causal relationships between time series can be inferred from their temporal relationships (Granger, 1969). If past values of one time series improve predictions of current and future values of another, there is Granger causality (GC) from the first time series to the second. It is important to note that significant causality in one direction does not assure causality in the other.

Whilst GC is primarily a time-domain concept (Goebel et al., 2003), assessing connectivity in the frequency domain has several advantages: physiological and non-physiological noise factors have characteristic spectral characteristics whose effects can be minimised; and endogenous BOLD oscillations are maximal within distinct frequency bands (Cordes et al., 2000). With this in mind, the time series of all six components of interest were bandpassed between 0.05 and 0.15 Hz.

Generalised partial directed coherence (GPDC) is a frequency-based measure of functional connectivity, which has recently been applied to fMRI data (Havlicek et al., 2010; Sato et al., 2009; Shim et al., 2013; Silfverhuth et al., 2011). GPDC can evaluate the direction of information flow similarly to alternative frequency-based approaches, such as the directed transfer function and relative power contribution, and is equivalent to these measures in bivariate cases, but holds a significant advantage when assessing multivariate data, as it can discern direct and indirect influences on causal relationships by partialling out the effects of additional time series (Sato et al., 2009).

The formalised description of the GPDC approach below follows the elegant previous conceptualisations laid out elsewhere (Baccala and Sameshima, 2001; Sato et al., 2009). Inferences of Granger causality are made using vector autoregressive modelling. Supposing **Y***_t_* is a multidimensional time series made up of *k* signals such that

(1) $$Y_{t}=\left\lfloor \begin{array}{c} y_{1t}\\ y_{2t}\\ \vdots \\ y_{\mathrm{kt}} \end{array}\right\rfloor ,\;t=1,2,\ldots T$$

the vector autoregressive model can be calculated by

(2) $$Y_{t}=v+\sum_{t=1}^{p}A_{l}Y_{t-1}+\varepsilon_{t}$$

in which **v** is a vector of constants and **ε***_t_* is a vector of random disturbances. The matrices **A***_l_* (*l* = 1, …, *p*) in turn represent

(3) $$A_{l}=\left[\begin{array}{cccc} a_{11}^{\left(l\right)} & a_{12}^{\left(l\right)} & \cdots & a_{1k}^{\left(l\right)}\\ a_{21}^{\left(l\right)} & a_{22}^{\left(l\right)} & \cdots & a_{2k}^{\left(l\right)}\\ a_{31}^{\left(l\right)} & a_{32}^{\left(l\right)} & \cdots & a_{3k}^{\left(l\right)}\\ \vdots & \vdots & \ddots & \vdots \\ a_{k1}^{\left(l\right)} & a_{k2}^{\left(l\right)} & \cdots & a_{\mathrm{kk}}^{\left(l\right)} \end{array}\right]$$

and the element *a*_*ij*_^(*l*)^ (*i* = 1, …, *k*; *j* = 1, …, *k*) is the causality coefficient from the time series *y_jt_* to the series *y_it_*. The vector **ε***_t_* has a covariance matrix given by

(4) $$\sum =\left[\begin{array}{cccc} \sigma_{11}^{2} & \sigma_{12}^{2} & \cdots & \sigma_{1k}^{2}\\ \sigma_{21}^{2} & \sigma_{22}^{2} & \cdots & \sigma_{2k}^{2}\\ \sigma_{31}^{2} & \sigma_{32}^{2} & \cdots & \sigma_{3k}^{2}\\ \vdots & \vdots & \ddots & \vdots \\ \sigma_{k1}^{2} & \sigma_{k2}^{2} & \cdots & \sigma_{\mathrm{kk}}^{2} \end{array}\right].$$

Under the assumption that **ε***_t_* is zero, using all information until time (*t* − 1), **Y***_t_* can be predicted by

(5) $${\hat{Y}}_{t}=v+\sum_{l=1}^{p}A_{l}Y_{t-1}.$$

In this vector autoregressive model, *y_jt_* can be considered to Granger-cause *y_it_* if the coefficient *a*_*ij*_^(*l*)^ is nonzero for a particular value of *l*.

GPDC is the frequency domain representation of Granger causality (Baccala and Sameshima, 2001; Takahashi et al., 2010), which incorporates variance stabilisation with the effect of improving the decision error rate of classified causal influence (Baccala et al., 2006; Sato et al., 2009). GPDC from time series *j* to time series *i* at frequency *λ* is defined by:

(6) $$\pi_{\mathrm{ij}}\left(\lambda \right)=\frac{a_{\mathrm{ij}}\left(\lambda \right)\frac{1}{\sigma_{i}}}{\sqrt{{\sum_{i=1}^{k}\left|a_{\mathrm{ij}}\left(\lambda \right)\right|}^{2}\frac{1}{\sigma_{i}^{2}}}}$$

where

(7) $$a_{\mathrm{ij}}\left(\lambda \right)=\delta_{\mathrm{ij}}-\sum_{l=1}^{p}a_{\mathrm{ij}}^{\left(l\right)}\exp\left(-\frac{2\pi }{\lambda \sqrt{1}}\right)$$

for *δ_ij_* = 1 if *i* = *j* and 0 otherwise. The square modulus of the GPDC value from the *j*-th time series by the *i*-th series can be understood as the proportion of the power spectra of the *j*-th time series sent to the *i*-th series after accounting for the effects of the other series (Sato et al., 2009). GPDC is bounded between 0 and 1, with the former representing an absence of functional connectivity from the *j*-th time series to the *i*-th time series and the latter indicating strong connectivity between these signals.

Here, GPDC was evaluated between all-pairwise component time courses using the Functional Network Connectivity toolbox (FNC; version 2.3; http://mialab.mrn.org/software/fnc/). Crucial to the accuracy of multivariate autoregressive procedures, such as GC analysis using GPDC, is the generation of the transfer matrix to which the data is fitted (Bianchi et al., 2013). In light of recent demonstrations of advantages for Bayesian Information Criterion (BIC) over Akaike Information Criterion (AIC) approaches (Hemmelmann et al., 2009; Porcaro et al., 2009) due to order-overestimation in AIC models, model order was estimated in the current analysis using a group-level BIC estimate.

This analysis produced bi-directional indices of GPDC for each pairwise combination of components in 0.002 Hz frequency bins between 0.05 and 0.15 Hz, with this bin resolution determined by FNC toolbox algorithms on account of the repetition time of the fMRI data and the extent of the frequency band of interest. Decomposing the data in this way was done to afford greater spectral specificity to the results in light of previous observations that fMRI functional connectivity varies across frequencies (Salvador et al., 2005). To investigate between-group differences in GPDC in a manner which utilised this multidimensional set of interdependencies, several analyses were conducted. In an initial exploratory analysis, Kruskal–Wallis tests were conducted using a 95% threshold to assess between-group differences in GPDC magnitude for each pairwise combination of components within each frequency bin. A spectral-clustering criterion was used to minimise Type-1 errors, whereby between-group differences were reported as significant only when differences at the 95% threshold were observed for five neighbouring frequency bins.

To assess whether overall GPDC-connection strength distribution differed by study group, the percentage of connections was calculated as a function of GPDC magnitude for VPT-born individuals and controls independently. On the basis of the resultant histograms (see Results), connections were categorised in three connectivity windows (CWs) as: low strength (0.05 < GPDC < 0.20); mid-strength (0.20 < GPDC < 0.35); or high strength (GPDC > 0.35). Between-group differences were then evaluated using a leave-one-out permutation approach by which multiple Kruskal–Wallis tests were used to assess differences in percentage of connections and mean GPDC at each connection strength. Effects reliable in more than 98.3% of permutations were considered significant; this threshold signifies Bonferroni correction of the conventional 95% threshold, to take into account the three windows of investigation.

To investigate between-group differences in the topology of the network of neurocognitive networks, linear discriminant analysis (LDA) was conducted. LDA can be used to identify feature sets which permit categorical dissociation by defining a linear summary of multidimensional data, and has been used previously to classify individuals on the basis of traumatic brain injury (Castellanos et al., 2010). Here, LDA was conducted on binarised matrices denoting connection topology according to thresholded GPDC. Each matrix featured bi-directional connections between all components. Accuracy of this procedure at classifying individuals by study group was assessed at varying GPDC thresholds, with the rationale that the identification of a GPDC threshold which successfully classifies individuals by group denotes a clinically useful marker of VPT-birth associated dysfunction. Correct classification on the basis of Fisher discriminant value compared to the projected hyperplane was calculated for each participant; and validated using a leave-one-out strategy for all possible participant permutations. Discriminant accuracy is defined here as the product of population accuracy and Mahalonobis distance (Castellanos et al., 2010). The projection weights can be reasonably interpreted as the relative contribution of each path to the discriminant function that best separates the groups (Ecker et al., 2010), permitting us to identify paths particularly contributing to the discrimination of the physiology of term-born and VPT-born individuals. However, it should be emphasised that due to the multivariate characteristic of classifying methods, each path in the projecting space should be interpreted in the context of the entire discriminating pattern and should not be considered in isolation (Ecker et al., 2010).

To complement the current GC analyses, a functional connectivity analysis was additionally performed by means of zero-lagged correlation between the time courses of activity of the neurocognitive networks and is presented as Appendix A.

### 2.9 GPDC, executive function and perinatal risk factors

It has recently been observed that preterm-birth related impairments in executive function preferentially persist into adulthood (Allin et al., 2011; Nosarti et al., 2007); and that impairments in executive function result from disruption of the coordination of activity across large-scale functional brain networks (Repovs et al., 2011). With the motivation of further examining the putative relationship between executive function and network coordination, whilst assessing the moderating effects of factors associated with preterm birth, several additional analyses were conducted.

First, to evaluate whether network coordination predicted executive function after timely and uncomplicated birth, a linear regression analysis was performed for the term-born group data using executive function as the dependent variable and mean GPDC in LDA-determined paths as the independent variable. Next, to investigate the putative relationship between these variables in VPT-born individuals, a corresponding linear regression analysis was conducted in this group. Finally, to test the hypothesis that perinatal factors play a modulatory role on the strength of this relationship, the moderating effects of GA and neonatal US classification on the pathway from GPDC to executive function were assessed using the PROCESS macro expansion (Hayes, 2013) for SPSS (SPSS Inc., USA). The reported collinearity between GA and US classification precluded concomitant investigation of their effects using regression. Instead, two independent moderation analyses were therefore performed. Moderation analysis within PROCESS utilises ordinary-least squares regression and permits the flexible evaluation of diverse statistical interdependencies. Here, the predictive effects on executive function of: (i) GPDC (the independent variable); (ii) US classification or GA (the moderating variable); and (iii) the interaction of GPDC and the perinatal moderator were modelled. Hypothesis testing was performed on the basis of each regression coefficient and its standard error calculated over 1000 randomly bootstrapped samples, with significance ascribed using percentile-based bootstrap confidence intervals and related α-levels Bonferroni corrected to .025 to reflect the number of perinatal factors investigated.

(In an attempt to elucidate the primacy of these perinatal factors on GPDC-derived network topology a series of complementary LDA investigations were conducted with the rationale that the factor shown to be associated with the most robust deviation from the normative data could be reasonably adjudged to exert the most deleterious effect. These analyses are presented as Appendix B.)

## 3 Results

### 3.1 Head movement

Table 2 presents the group-averaged head-movement characteristics. The degree of head movement did not significantly differ between the study groups in any calculated metric.

### 3.2 Components

Fig. 1 illustrates GOF scores for all 44 components with each of the six subnetworks of interest. For five of these, the component with the greatest GOF score was selected for subsequent connectivity analysis. Visual inspection of the best-fit striatal salience network component suggested that this component was primarily focused in ventricular spaces. This was confirmed by an additional GOF evaluation, whereby this component was found to be the best-fit component with a cerebrospinal fluid mask. (Please refer to Appendix C for further details.) The component with the second-highest GOF score with the striatal salience network mask was therefore selected for connectivity analysis.

**Fig. 2:**
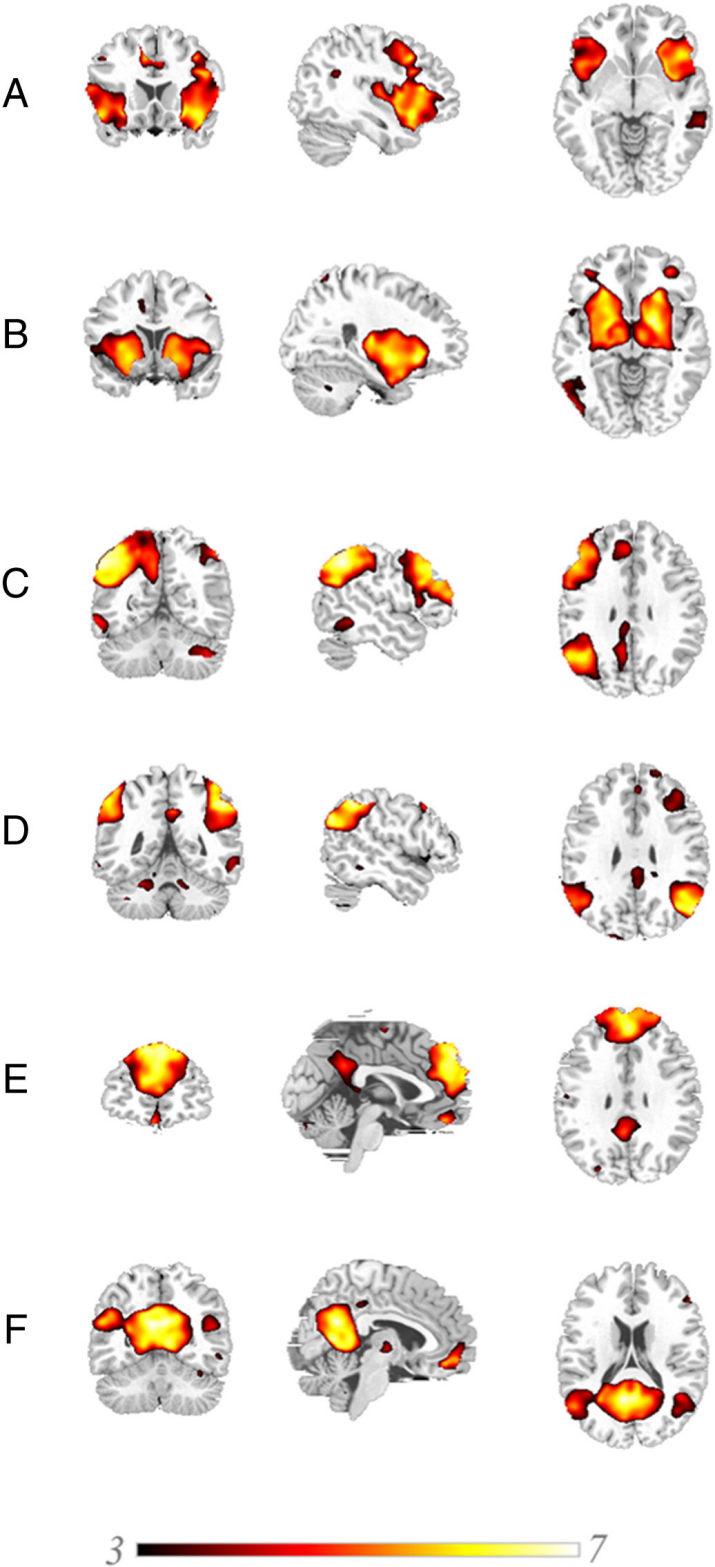
Components of interest, showing (A) insular salience network (B) striatal salience network; (C) left central executive network; (D) right central executive network; (E) frontal default mode network; and (F) posterior default mode network. Results depicted represent clusters with significant positive loadings (P < .05, family-wise error corrected) on the basis of one-sample T-tests including all study participants. Results are overlaid on standardised T_1_-weighted image and scaled according to the T-value colour-bar shown.

The six components identified on the basis of their spatial concordance with well-studied large-scale distributed networks therefore included: (A) the insular portion of the salience network, focused on bilateral inferior frontal gyrus and insula; (B) the striatal component of the salience network, focused on the putamen bilaterally; (C) the left-hemispheric constituents of the CEN, with strong loadings in left-hemispheric inferior parietal lobule and dorsolateral prefrontal cortex (PFC); (D) the right-hemispheric CEN, with strong loadings in right-hemispheric dorsolateral parietal and frontal regions; (E) the frontal portion of the DMN, with maximal loadings in medial and superior PFC; and (F) the posterior portiion of the DMN, focused on posterior cingulate gyrus and bilateral angular gyrus. These components are depicted in Fig. 2 and their grey-matter foci presented in Table 3.

### 3.3 Spatial differences between VPT-born and term-born group components

Analysis of between-group within-component effects revealed that for the right CEN component only a region of the right anterior insula exhibited significantly reduced loadings in the VPT-born individuals compared to controls. Characteristics of this effect are shown in Table 4.

### 3.4 Spectral differences between VPT-born and term-born group components

**Fig. 3:**
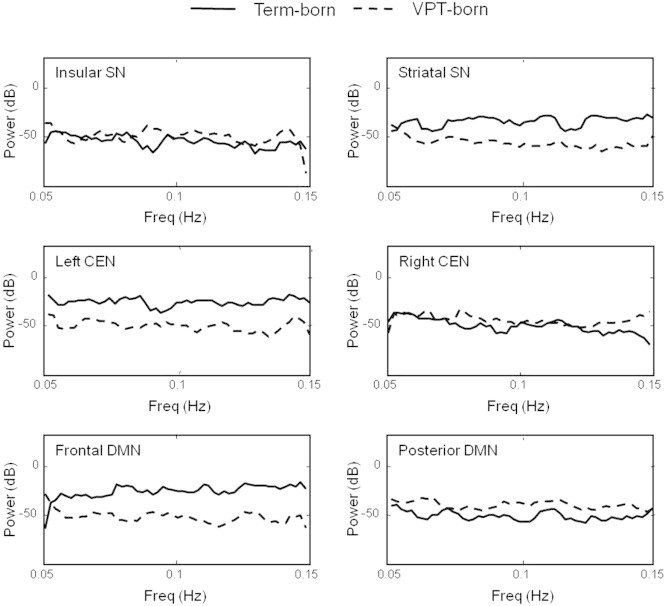
Group-averaged periodograms, depicting power spectrum density for the six components of interest. SN, salience network; CEN, central executive network; DMN, default mode network; VPT, very preterm.

Broadband power (between 0.05 and 0.15 Hz) for each component is displayed in Fig. 3. Mean power was not significantly different between groups for any component.

### 3.5 Generalised partial directed coherence

Between-group comparisons of GPDC within each frequency bin and for each pairwise combination of components revealed path- and frequency-specific reductions in GPDC in the VPT-born compared to the term-born individuals. These findings are presented in Table 5. No significant increases in GPDC were observed in the VPT-born group compared to the term-born controls.

**Fig. 4:**
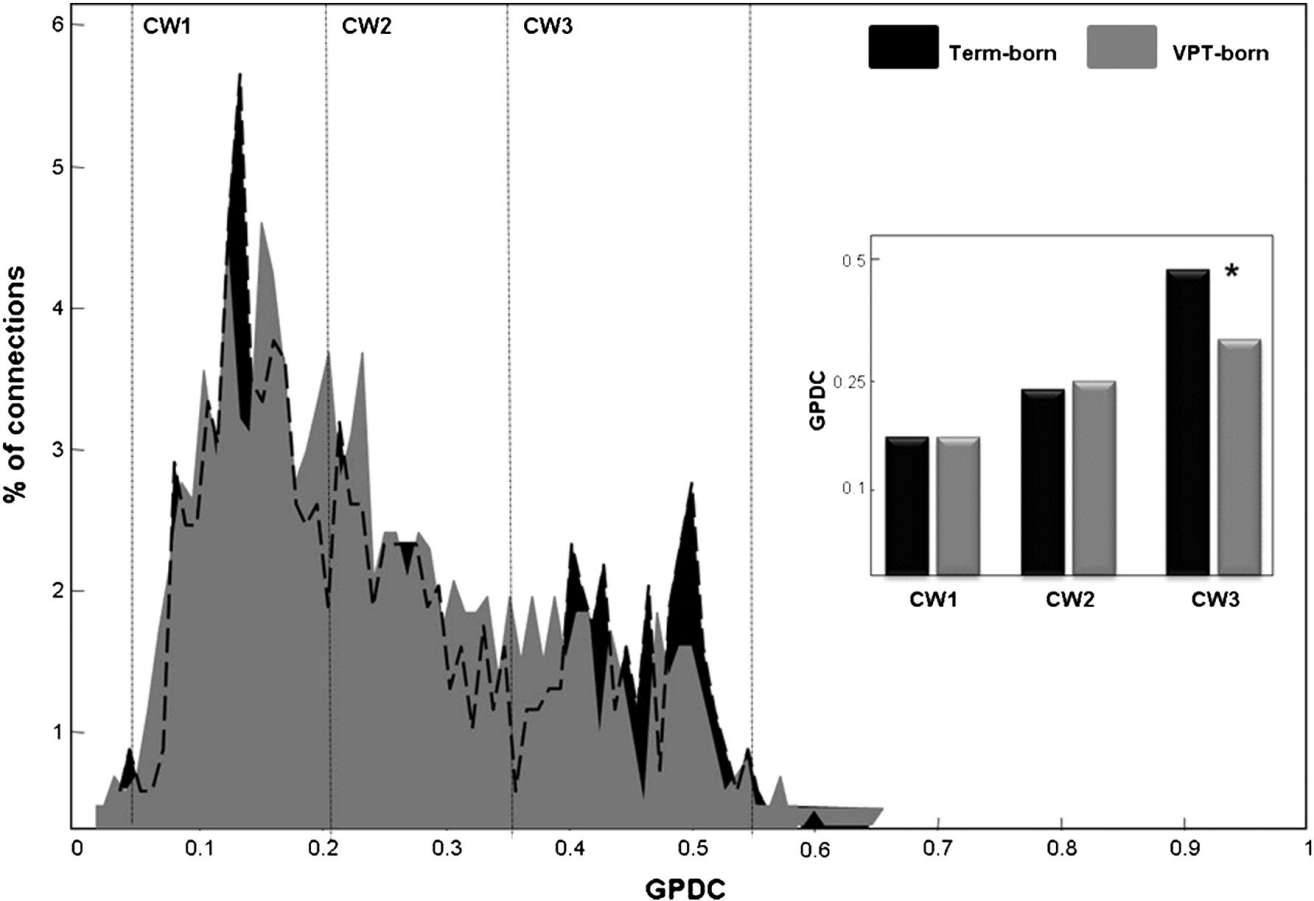
Group-averaged histograms showing the percentage of connections as a function of generalised partial directed coherence (GPDC). Dotted black line shows term-group distribution when hidden by VPT-group results. Other vertical lines represent boundaries for low (0.05–0.20), mid (0.20–0.35) and high (0.35–0.55) GPDC. Inset: Bar diagram shows grand-average connection strength for each connectivity window (CW). Asterisk denotes significant between-group difference in CW3.

Fig. 4 presents GPDC distributions for both study groups. Low-strength connections were the most numerous in both groups (VPT: 43.4%; term-born: 43.6%); and non-significant between-group differences were noted in the percentage of low- or mid-strength connections or the mean GPDC within these windows. By contrast, high-strength connections were robustly more numerous in the term-born group as compared with the VPT-born group (VPT: 22.02%; term-born: 26.2%, P = .0016). Furthermore, mean GPDC within the high-strength connection window was significantly greater in term-born as compared with VPT-born individuals (VPT: 0.43 ± 0.05 (mean GPDC ± standard deviation); term-born: 0.45 ± 0.05, P = 0.0097).

### 3.6 Linear discriminant analysis

**Fig. 5:**
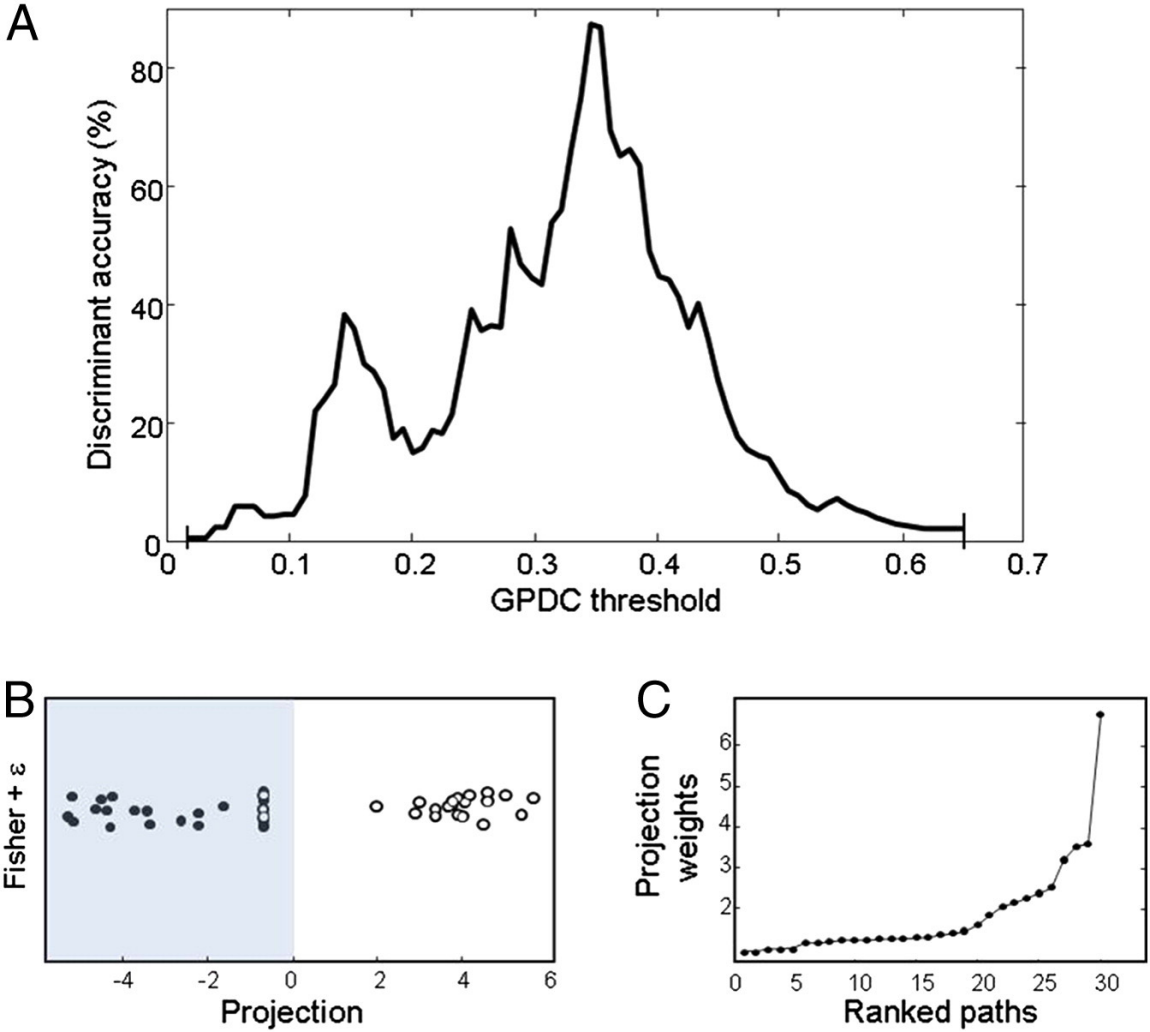
Classification by whole-network topology, using linear discriminant analysis of binarised networks according to variable generalised partial direct coherence (GPDC) thresholds, showing: (A) classification accuracy as a function of GPDC threshold; (B) scatterplot of classification at a GPDC threshold of 0.35; and (C) ranked LDA weights for each path for classification at a GPDC threshold of 0.35.

Fig. 5 presents the results of the LDA performed to assess group-classification accuracy on the basis of network topology as a function of weighting threshold. Classification accuracy was critically improved by varying the GPDC threshold, reaching a maximum accuracy (86%) at 0.35 (Fig. 5A); here, all control participants are correctly classified and only 4 of 29 VPT-born individuals are misclassified (Fig. 5B). LDA also provides path-specific weights for the discrimination. Fig. 5C shows the weights for all 30 paths for the LDA using a GPDC-threshold of 0.35. These weights permit identification of the most pertinent paths for the topological discrimination of VPT- and term-born individuals.

**Fig. 6:**
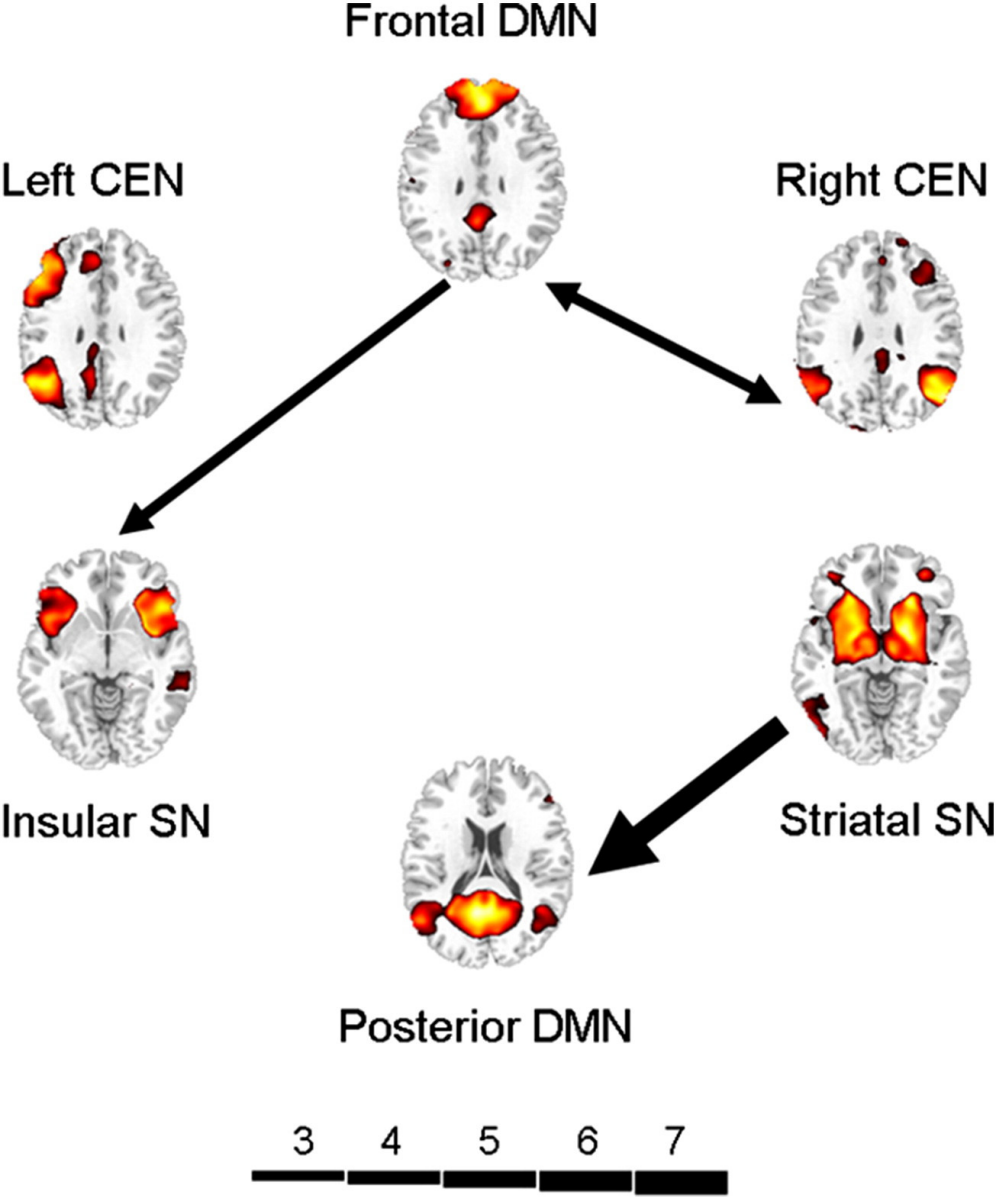
Pathways influential for accurate classification, showing critical pathways by projection weight. Arrow breadth denotes path-specific weights from linear discriminant analysis according to the scale depicted. DMN, default mode network; CEN, central executive network; SN, salience network.

Fig. 6 shows the path-specific weights associated with the most accurate classification, and suggests that the path from striatal SN to posterior DMN was particularly useful in differentiating the groups. Also shown to be important were reciprocal connections between frontal DMN and right CEN.

### 3.7 The relationship between GPDC and executive function

Executive function was not significantly predicted by GPDC in either study group considered as a whole. However, the moderation analysis revealed that in the VPT-born group executive function was significantly predicted by US classification (β = − 1.70 ± 0.69, T = − 2.49, P = .020) and further that there was a significant interaction on executive function between GPDC and US classification (β = 6.72 ± 2.74, T = 2.45, P = .023). Similarly, in the analysis assessing the moderating effects of GA, GA was observed to significantly predict executive function (β = 0.67 ± 0.23, T = 3.00, P = .007) and an interaction on executive function was observed between GA and GPDC (β = − 2.80 ± 0.97, T = − 2.88, P = .008). Furthermore, accounting for GA produced a significant effect of GPDC on executive function in the VPT-born group (β = 83.45 ± 28.53, T = 2.92, P = .008). Stratification by GA revealed that the most robust relationship between GPDC and executive function was observed in the low GA individuals (β = 8.20 ± 3.40, T = 2.41, P = .024).

## 4 Discussion

The primary aim of the current study was to delineate differences in resting-state functional connectivity within and between three important and robust neurocognitive brain networks in VPT-born adults relative to term-born controls. No significant between-group differences were noted in broadband component power. Analysis of regionally-specific component loadings revealed no significant differences between VPT- and term-born adults in terms of network spatial focus for five of the six components. The sole significant between-group difference was observed in the right CEN component where term-born individuals exhibited significantly greater loadings in the right anterior insula. However, since this region was not a significant focus of the right-CEN component, but was instead included with the insular-SN component (Table 3), this finding is likely to reflect a between-network rather than within-network difference. The general consistency in cross-group component focus indicates that the selected components were reliably evident in both groups and serves as useful validation for their subsequent targeted evaluation. Furthermore, it demonstrates that in adulthood RSNs are not for the most part distinguishable in their spatial characteristics between preterm- and term-born individuals. This supports the findings of previous investigation of RSN spatial focus in infancy, where differences in RSN location were small and few (Damaraju et al., 2010).

Despite the general consistency of within-network focus and spectral power, several important differences were observed in between-network connectivity as indexed by GPDC. First, viewing all frequency bins for all pairwise combination of components, there was a robust reduction in the number of high-strength (GPDC > 0.35) connections in VPT-born individuals; that is, fewer networks were strong predictors of future activity in other networks in the VPT-born group compared to controls. This implies a functional disconnect in the adult VPT-born brain. In other words, these findings suggest less robust inter-network coordination in these individuals. Reduced temporal anti-correlation between DMN and exogenously-directed attention networks has been suggested as a useful index of compromised brain function in association with psychiatric illness (Broyd et al., 2009; Buckner et al., 2008; White et al., 2010) and sleep deprivation (De Havas et al., 2012). Our current findings are consistent with VPT-birth related disruption to this mechanism. This may provide explanation for the increased occurrence of attentional lapses in young preterm-born adults (Nosarti et al., 2007), since attentional lapses can accordingly be explained in terms of ineffectual DMN suppression during active information processing (Shulman et al., 2007).

The between-group analysis of GPDC at specific frequencies for each pairwise combination of components permitted greater insight into the between-network connections preferentially affected by preterm birth. Significantly greater GPDC was shown by the term- compared to the VPT-born group from insular SN to both frontal DMN (0.0736–0.0814 Hz) and posterior DMN (0.1124–0.1221 Hz), from striatal SN to insular SN (0.1124–0.1221 Hz), from right CEN to striatal SN (0.1221–0.1298 Hz) and from posterior DMN to left CEN (0.0911–0.0988 Hz). The presence of SN components in the majority of these relationships provides evidence that abnormalities in resting-state BOLD fluctuations in the basal ganglia (Damaraju et al., 2010) endure into adulthood.

Our findings of reduced functional connectivity between neurocognitive networks in preterm-born individuals extend previous knowledge on two important fronts. First, they imply that resting-state abnormalities evident during infancy (Doria et al., 2010; Fransson et al., 2007) persist into adulthood; and second, they demonstrate that functional connectivity abnormalities are systemically pervasive. Preterm-born adolescent reports of aberrant functional connectivity between language centres imply the development of alternate compensatory functional pathways (Gozzo et al., 2009; Myers et al., 2010). Here, we show deficits to the system proposed to be fundamentally responsible for task-appropriate attentional focus (Menon, 2011). It is feasible that the abnormalities found currently underlie wide-ranging functional deficits. Specifically, the VPT-born sample of this study exhibit EF impairments in addition to widespread reduction in between-network GPDC. Nevertheless, since preterm-born individuals exhibit recovery and preservation of diverse aspects of cognitive function (Hack, 2009; Hack et al., 2004; Ment et al., 2003), it remains a central challenge to determine how the longitudinal cerebral trajectories of specific functional systems (and their interaction) relate to cognition and behaviour in these individuals.

The use of classification by connectivity is an area of burgeoning scientific interest (see, for example, Anticevic et al., 2013; Uddin et al., 2013), and of huge potential clinical relevance in light of the numerous disconnection syndromes evident in psychiatry and neurology. Here, the potential utility of aberrant between-network GPDC as a marker of anomalous function associated with VPT birth was evaluated using LDA. This analysis demonstrated that between-network topology (on the basis of GPDC) successfully classified individuals by group, and also that the accuracy of this classification was improved by setting the inclusion threshold for connections within the network to high but prevalent connection strengths.

The LDA also demonstrated that the most powerful connection for accurately classifying participants was the path from striatal SN to posterior DMN, which implicates this path as a source of pathology in the VPT-born group. Disturbances to SN function can result in impaired bottom-up detection of salient events and reduced functional coupling between insula and ACC, which in turn disrupts rapid communications with the motor system (Menon, 2011). Whilst these impairments particularly highlight the role that the SN plays in facilitating rapid attentional and behavioural responses to external cues (Dosenbach et al., 2006), they hold no less importance in relation to SN-DMN interactions. Conceptually, SN-produced disengagement of DMN processes is as critical as CEN engagement to exogenously-focused information processing; further, SN is likely to elicit contextually-relevant engagement of DMN for introspective and mnemonic processes (Sridharan et al., 2008). This work, in providing robust evidence that coordination between SN regions and DMN regions is compromised in VPT-born adults, highlights a potential pathophysiological mechanism for the deficits in performance IQ, memory and executive function observed in young VPT-born adults (Allin et al., 2011; Hille et al., 2007; Nosarti et al., 2007).

A crucial premise of this work is that cognitive, educational and behavioural problems persist into adulthood in VPT-born individuals. Despite non-significant differences in IQ as compared with term-born control subjects, deficits in executive function were seen in VPT-born group. This finding supports observations at approximately 23 years from an overlapping sample of the same cohort (Nosarti et al., 2007) and suggests lasting impairments in this domain. Interestingly, the analyses investigating the moderating effects of perinatal risk factors (GA and US classification) on the relationship between GPDC and executive function revealed that both GA and US significantly predicted executive function. Furthermore, the significant interactions between both perinatal factors and GPDC on executive function, and the finding that the relationship between GPDC and executive function was more robust in low-GA VPT-born individuals, present the pathway from inter-network GPDC to executive function as especially mechanistically relevant for these individuals. This suggests a possible failure to develop a diverse repertoire of physiological approaches to meet environmental challenges in prematurely-born individuals, leaving them susceptible to executive-function decrements if the inter-network communication system breaks down.

Although of comparable size with other recent RSFC investigations of psychopathology (for example, Manoliu et al., 2013; Shin et al., 2013), the current sample is relatively small and current results may not be generalisable to other VPT-born samples defined by different neonatal characteristics. Future investigation of larger samples will improve the power to detect relationships between functional imaging measures, their structural correlates and cognitive and psychiatric outcome data. Another related factor is that since morbidity associated with preterm birth (including germinal matrix haemorrhage, cavitary white matter disease, and permanent lung damage) has drastically diminished over the 30 years since the current sample was born (Hack, 2007), it is likely that the phenotypic characteristics (for example, RSFC) of this population in adulthood will continue to evolve in line with these improvements.

Given that those VPT-born individuals born with the lowest gestational age tended also to be those who received an abnormal neonatal ultrasound classification, it is difficult to disentangle the effects of premature birth per se from those accountable to perinatal brain injury in association with premature birth. This inter-relatedness of risk factors motivated the additional LDA investigations presented in Appendix B, although it is stressed that independent investigation of these factors is likely underpowered in the current sample on account of the size of the sub-samples involved. The observation that the most accurate classification of stratified groups occurred when assessing network topology in individuals with abnormal neonatal ultrasound classification and term-born individuals (93%) tentatively implies that brain injury precipitates the most robust deviation from normative patterns of functional connectivity; although the possibility that gestational age and brain injury interact cannot be discounted.

It is additionally noteworthy that the validity of inferring directional information flow from Granger-causality based fMRI metrics is a subject of active and intense debate. As lag-based procedures can be influenced by neurally- and haemodynamically-derived inter-regional lags, it is important to control adequately for the latter (Smith et al., 2011; Smith et al., 2012). In principle, this can be achieved via the acquisition of concurrent electrophysiological data (David et al., 2008); but this is in turn practically problematic in clinical groups, and integration of the resultant complementary signals is not trivial. Alternatively, analytic improvements such as modelling variation in signal-dependent noise can potentially refine Granger-causality estimation (Luo et al., 2013). Nevertheless, whilst there is undoubtedly scope for methodological improvement, Schippers et al. (2011) recently reported good sensitivity and specificity of current Granger-causality based procedures to neural causal influences in simulation; and recent findings (for example, Palaniyappan et al., 2013) demonstrate the potential utility of non-zero-lagged procedures for improving our understanding of the physiological determinants of specific clinical symptoms. Furthermore, the divergent results of our own GPDC and zero-lagged analyses (Appendix A) demonstrate additional perspectives offered by non-zero-lagged functional-connectivity analysis.

This study, the first to our knowledge of resting-state functional connectivity in VPT-born adults, demonstrates widespread reduction in the causal interdependencies of large-scale neurocognitive networks in these individuals whilst at rest, and therefore provides a potential physiological substrate for the cognitive deficits that these individuals display. Connectivity between the SN and DMN was particularly diminished, although this disturbance was seen in the context of a generalised between-network dysconnectivity. These findings have implications not only for our understanding of the long-lasting effects of preterm birth on the systems fundamentally responsible for the coordination of brain activity, but also for the development of therapeutic practices which may lessen liability to cognitive impairments and psychiatric illness in this growing population.

## Figures and Tables

**Table 1:** Sample characteristics. Frequencies and mean values with bracketed standard deviations.

	Term-born group (N = 23)	VPT-born group (N = 29)
*Demographic information*		
Age at testing (years)	27.55 (2.22)	28.59 (2.04)
Sex (males/females)	10/13	12/17

*Cognitive assessment*		
Full-scale IQ	116.00 (12.20)	106.25 (11.59)
Verbal IQ	114.70 (12.16)	105.90 (12.74)
Performance IQ	114.70 (13.06)	105.35 (12.45)
Executive function (Hayling scaled)	6.60 (1.05)	5.78 (1.40)

VPT, very preterm; IQ, intelligence quotient.

**Table 2:** Group-averaged characteristics of head motion. Standard deviations are given in brackets.

Metric	Group	
	Term-born individuals	VPT-born individuals
Mean motion	0.060 (0.025)	0.078 (0.034)
Maximum motion	0.341 (0.245)	0.474 (0.434)
Number of movements	34.56 (37.38)	60.16 (46.85)
Rotation	8 × 10^− 3^ (5 × 10^− 3^)	0.001 (3 × 10^− 3^)

VPT, very-preterm.

**Table 3:** Grey-matter foci for the six components of interest. Reported voxels are within clusters significant at a family-wise error corrected P-threshold of .05.

Component	Location (Brodmann Area)	Talairach coordinates			Peak voxel T-statistic
		x	y	z	
Insular SN	Inferior frontal gyrus (13)	− 32	14	− 12	6.10
	Inferior frontal gyrus (13)	− 40	26	10	5.73
	Inferior frontal gyrus (13)	− 44	26	− 2	5.57
	Insula (13)	− 40	6	2	5.38
	Insula (13)	44	16	− 2	4.90
	Superior temporal gyrus (38)	48	14	− 10	5.77
	Anterior cingulate gyrus (24)	0	22	36	5.40
Striatal SN	Putamen	32	− 14	6	7.31
	Putamen	− 24	22	− 10	7.18
	Insula (13)	− 46	12	0	5.33
	Middle occipital gyrus (37)	56	− 70	0	5.73
	Fusiform gyrus (27)	38	− 56	− 16	5.52
Left CEN	Inferior parietal lobule (40)	− 46	− 52	46	6.41
	Inferior parietal lobule (40)	40	− 60	54	6.56
	Superior frontal gyrus (8)	− 34	16	56	6.13
	Superior frontal gyrus (8)	34	22	54	5.35
	Middle frontal gyrus (10)	− 38	46	− 6	6.26
	Middle frontal gyrus (10)	40	22	52	5.33
	Medial frontal gyrus (8)	2	40	48	5.33
	Cingulate gyrus (31)	− 2	− 28	44	6.61
	Cingulate gyrus (31)	− 4	− 46	38	5.40
	Inferior temporal gyrus (20)	− 62	− 28	− 20	5.34
	Middle temporal gyrus (21)	50	− 64	26	5.34
	Cerebellum: posterior lobe	32	− 68	− 38	5.69
	Cerebellum: anterior lobe	16	− 56	− 30	5.59
	Cerebellum: posterior lobe	20	− 82	− 32	5.49
Right CEN	Superior parietal lobule (7)	32	− 66	58	7.23
	Middle frontal gyrus (8)	36	26	52	7.17
	Inferior frontal gyrus (9)	56	6	30	5.33
	Precuneus (7)	6	− 54	40	5.80
	Parahippocampal gyrus (36)	14	− 36	2	5.41
	Middle temporal gyrus (21)	62	− 50	− 10	5.85
	Middle temporal gyrus (21)	− 58	− 2	− 10	5.83
	Cerebellum: posterior lobe	− 10	− 84	− 30	6.36
	Cerebellum: posterior lobe	− 30	− 64	− 40	5.82
Frontal DMN	Superior frontal gyrus (8)	8	54	44	6.78
	Superior frontal gyrus (9)	28	48	38	5.48
	Medial frontal gyrus (9)	− 2	46	32	6.37
	Medial frontal gyrus (9)	10	58	20	6.23
	Middle frontal gyrus (10)	− 26	58	26	5.54
	Anterior cingulate gyrus (32)	4	32	22	5.39
	Orbital gyrus (11)	0	44	− 20	5.50
Posterior DMN	Posterior cingulate gyrus (30)	22	− 68	10	5.38
	Posterior cingulate gyrus (31)	− 4	− 58	22	6.68
	Posterior cingulate gyrus (23)	2	− 36	40	5.81
	Medial frontal gyrus (11)	4	56	− 10	6.11
	Angular gyrus (39)	− 42	− 76	30	6.48
	Angular gyrus (39)	40	− 70	30	5.95
	Parahippocampal gyrus (36)	− 26	− 16	− 26	6.68
	Parahippocampal gyrus (36)	34	− 24	− 24	6.81
	Precuneus (19)	36	− 72	38	6.07
	Precuneus (19)	− 30	− 76	42	5.71
	Superior occipital gyrus (19)	− 36	− 82	30	6.54
	Thalamus	10	− 10	3	5.52
	Middle frontal gyrus (8)	30	20	48	5.49

SN, salience network; CEN, central executive network; DMN, default mode network.

**Table 4:** Between-group component spatial differences.

Component	Contrast	Location (Brodmann Area)	Talairach coordinates			Peak voxel T-statistic
			x	y	z	
Right CEN	Term > VPT	Insula (13)	48	14	5	4.89

**Table 5:** Significant path- and frequency-specific between-group differences in GPDC.

Path direction	Band (Hz)	Effect direction	Significance
Right CEN → Striatal SN	0.1221–0.1298	Term-born > VPT-born	P = .018
Striatal SN → Insular SN	0.1124–0.1221	Term-born > VPT-born	P = .030
Insular SN → Posterior DMN	0.1124–0.1221	Term-born > VPT-born	P = .019
Insular SN → Frontal DMN	0.0736–0.0814	Term-born > VPT-born	P = .026
Posterior DMN → Left CEN	0.0911–0.0988	Term-born > VPT-born	P = .003

CEN, central executive network; SN, salience network; DMN, default mode network; VPT, very-preterm.
