# Supplementary material for: Dysconnectivity of neurocognitive networks at rest in very-preterm born adults
Source: Neuroimage Clin. 2014 Jan 18;4:352–65. doi: 10.1016/j.nicl.2014.01.005 (PMC3930099; doi:10.1016/j.nicl.2014.01.005)
Supplement: Supplementary file 1 — Supplementary material. [file mmc1.doc]

**Appendix A: Assessment of zero-lagged correlation**

**Supplementary Methods**

A supplementary analysis was conducted to evaluate zero-lagged correlation in the current dataset using the individual-specific timecourses for each of the six subnetwork components having bandpass-filtered these series between 0.05 to 0.15 Hz in line with the generalised partial direct coherence (GPDC) analysis. Partial correlations were evaluated for each pairwise combination of subnetworks, while accounting for the effects of the four other subnetworks and the six realignment parameters on the signals of interest. This approach was considered to be conceptually similar to the multivariate procedure used to assess GPDC, which also accounted for the effects of the other timecourses of interest, although it is stressed that the zero-lag approach is undirected.

Individual-specific correlation coefficients were Fisher transformed to permit group-level statistical investigation with parametric tests. One sample T-tests were then conducted using 1000 bootstrapped samples for each pairwise combination of components to test the hypothesis that there was a significant zero-lagged association between the timecourses of these subnetworks of interest. Bootstrapping was performed with the aim of reducing the effects of individuals with outlying results. Finally, independent samples T-tests were conducted on the Fisher-transformed correlation coefficients (using the same bootstrapping procedure) to test the hypothesis that zero-lagged correlation differed between the very-preterm born individuals and the term-born control group. For both the one-sample and independent-samples T-tests, significance was ascribed according to a Bonferroni-corrected P-value of .0033 on account of the 15 pairwise combinations assessed. However, results nominally significant at an uncorrected P-value of .05 are also reported for illustrative purposes.

**Supplementary Results**

All significant whole-sample correlations are presented in Table A1. These included significant positive correlations between the central executive network (CEN) components and between the salience network (SN) components; as well as between the left CEN and both default mode network (DMN) components. Significant negative correlations were not observed between any subnetworks. Significant between-group differences are displayed in Table A2. These were less robust than the whole-sample effects and did not survive Bonferroni correction. Nominally significant between-group effects were observed in the association between frontal DMN and striatal SN; and in the relationship between right CEN and insular SN. In both instances, the very-preterm born individuals exhibited a more positive correlation coefficient than the term-born controls.

**Table A1. Significant whole-sample zero-lagged correlations.** Bracketed values denote standard deviations. Asterisks denote significant effects after Bonferroni correction (P<.0033). Other effects are nominally significant at an uncorrected P-value of .05.

| Pairwise combination | Fisher-transformed correlation coefficient | T-value | Bootstrapped 95% confidence interval | Bootstrapped P-value |
| --- | --- | --- | --- | --- |
| Frontal DMN-Right CEN | 0.095 (0.197) | 3.46 | 0.040-0.150 | .004 |
| Frontal DMN-Left CEN | 0.119 (0.208) | 4.13 | 0.063-0.178 | .001* |
| Right CEN-Striatal SN | 0.052 (0.178) | 2.09 | 0.006-0.104 | .050 |
| Right CEN-Insular SN | 0.106 (0.280) | 2.72 | 0.030-0.186 | .034 |
| Right CEN-Left CEN | 0.057 (0.122) | 3.39 | 0.025-0.091 | .003* |
| Right CEN-Posterior DMN | 0.052 (0.157) | 2.40 | 0.010-0.092 | .018 |
| Striatal SN-Insular SN | 0.127 (0.218) | 4.21 | 0.068-0.183 | .001* |
| Striatal SN-Posterior DMN | 0.157 (0.212) | 5.34 | 0.100-0.215 | .001* |
| Left CEN-Posterior DMN | 0.119 (0.171) | 5.02 | 0.075-0.170 | .001* |

CEN, central executive network; SN, salience network; DMN, default mode network.

**Table A2. Significant between-group differences in zero-lagged correlations.** Bracketed values denote standard deviations. Asterisks denote significant effects after Bonferroni correction (P<.0033). Other effects are nominally significant at an uncorrected P-value of .05.

| Pairwise combination | Fisher-transformed correlation coefficient | | T-value | Bootstrapped 95% confidence interval | Bootstrapped P-value |
| --- | --- | --- | --- | --- | --- |
|  | Term-born | VPT-born |  |  |  |
| Frontal DMN-Striatal SN | -0.10 (0.17) | 0.04 (0.21) | -2.58 | -0.234 to -0.029 | .040 |
| Right CEN-Insular SN | 0.01 (0.13) | 0.18 (0.34) | -2.50 | -0.300 to -0.048 | .023 |

CEN, central executive network; SN, salience network; DMN, default mode network.

**Supplementary Discussion**

The results presented in Table A1 highlight robust positive correlations within and between neurocognitive networks across the full-study sample. The prevailing patterns of positive correlation were not unexpected on account of the non-inclusion of global signal regression in the procedure for timecourse extraction . In fact, several of the most robust positive relationships were seen between both portions of the DMN and the other neurocognitive networks. Other strong relationships were observed between the two CEN subnetworks and the two SN networks, which validates further the selection of the components of interest and the focus of investigation on these neurocognitive networks. It is noted, however, that the zero-lagged correlation between frontal and posterior DMN features was not significant.

In light of the widespread reductions in functional connectivity found in the VPT-born individuals as compared with the term-born group during the current generalised partial directed coherence (GPDC) investigation, the reduced sensitivity of zero-lagged correlations to these physiological abnormalities is noteworthy and serves to justify the use of GPDC to investigate directional information flow within the current study.

**Supplementary References**

Fox, M.D., Snyder, A.Z., Vincent, J.L., Corbetta, M., Van Essen, D.C., Raichle, M.E., 2005. The human brain is intrinsically organized into dynamic, anticorrelated functional networks. Proc Natl Acad Sci U S A 102, 9673-9678.

Murphy, K., Birn, R.M., Handwerker, D.A., Jones, T.B., Bandettini, P.A., 2009. The impact of global signal regression on resting state correlations: are anti-correlated networks introduced? Neuroimage 44, 893-905.

**Appendix B**

**Supplementary Methods**

A series of complementary linear discriminant analyses (LDA) were conducted to investigate the effects on generalised partial directed coherence (GPDC) defined graph topology of gestational age (GA) and prenatal injury as identified with neonatal ultrasound (US). GPDC-graph topology was compared between VPT-born and term-born individuals using the original classification method (section 2.8). In contrast to the main analyses, here the VPT-group was stratified, which permitted investigation of classification accuracy within and between our main study groups. VPT-born individuals were independently stratified by: (i) ultrasound classification, as normal (n=13) or abnormal (including uncomplicated periventricular haemorrhage (UPVH), and periventricular haemorrhage and ventricular dilatation (PVH+DIL), n=16); and (ii) gestational age, as low (<30 weeks, n=13) or high (≥30 weeks, n=16). VPT-born individuals with abnormal neonatal ultrasound classification and lower gestational age were combined to represent a ‘high-risk VPT group’ and those with normal neonatal ultrasound classification and higher gestational age were combined to represent a ‘low-risk VPT group’.

**Supplementary Results**

Highest classification accuracy was achieved when discriminating individuals with abnormal US classification and term-born individuals (93%; see Figure B1). Classification accuracy for LDA between high-risk VPT-born individuals and term-born controls (92%) exceeded that of the LDA between low-risk VPT-born individuals and term-born controls (82%). Furthermore, within the VPT-born group high classification accuracy was achieved in the discrimination between normal and abnormal neonatal ultrasonography (92%).

**Supplementary Discussion**

While these analyses do not wholly dissociate the effects of gestational age and ultrasound-recorded brain injury, our findings suggest that ultrasound is a more useful predictor of abnormal neurophysiology.


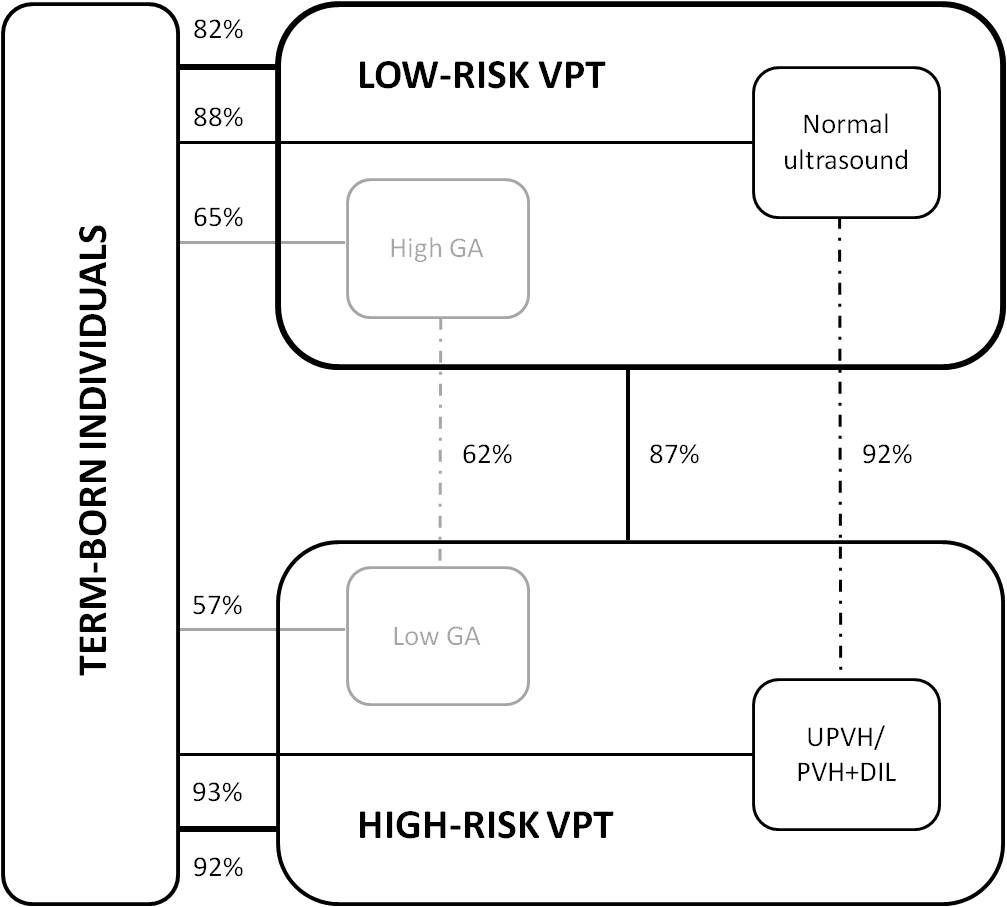


**Figure B1**. Classification accuracy of stratified linear discriminant analyses. Gestational age, GA; very-preterm, VPT; uncomplicated periventricular haemorrhage, UPVH; periventricular haemorrhage and ventricular dilatation, PVH+DIL.

**Appendix C**

**Supplementary Methods**

To test the hypothesis that the best-fit striatal salience network component was predominantly focused within cerebrospinal fluid (CSF), an additional goodness-of-fit (GOF) examination was performed using the same GOF calculation procedures as described in Methods. The CSF mask was calculated using the CSF template from SPM8 thresholded to include voxels whose likelihood of representing CSF exceeded .80.

**Supplementary Results**

Figure C1 depicts GOF ranked scores for each of the 44 component maps with the CSF mask. The best-fit striatal salience network component was also the best-fit CSF component. As the GOF score for CSF exceeded the GOF score for this component (but not the second best-fit striatal component), the second best-fit component was selected as the striatal salience network component for further investigation.


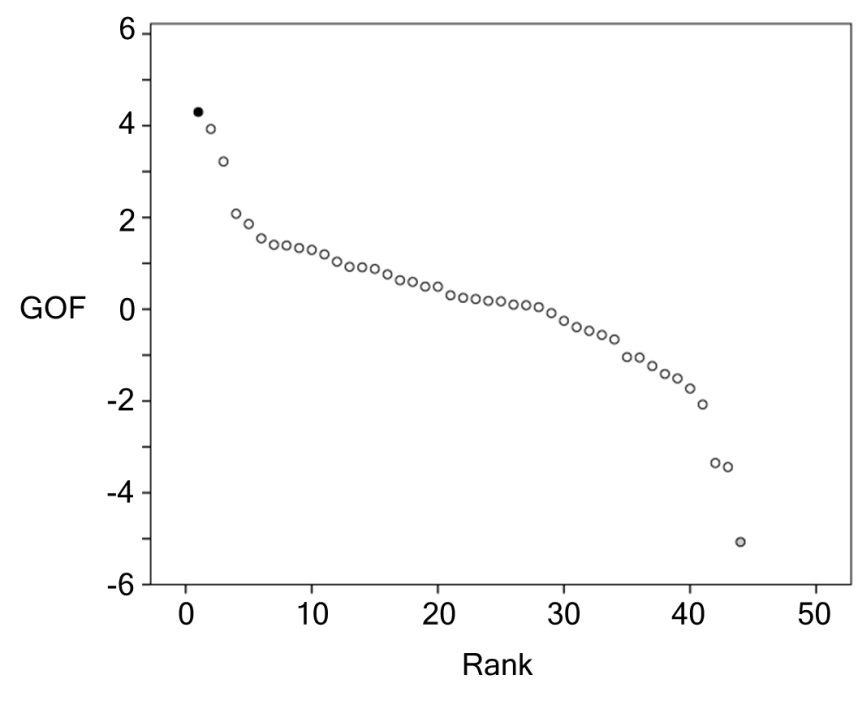


**Figure C1. Ranked goodness-of-fit (GOF) scores for each spatial component in relation to cerebrospinal fluid (CSF) mask.** Component shown in black is best-fit component for CSF mask and striatal salience network mask. Component shown in grey is second best-fit for striatal salience network and worst-fit for CSF mask.
